# Feasibility of Carbon Dioxide Storage Resource Use within Climate Change Mitigation Scenarios for the United States

**DOI:** 10.1021/acs.est.3c00790

**Published:** 2023-09-26

**Authors:** Yuting Zhang, Christopher Jackson, Nihal Darraj, Samuel Krevor

**Affiliations:** Department of Earth Science and Engineering, Imperial College London, Exhibition Road, London SW7 2BX, U.K.

**Keywords:** growth modeling, CO_2_ storage, growth rates, storage resource requirement, United States, net zero, climate change mitigation

## Abstract

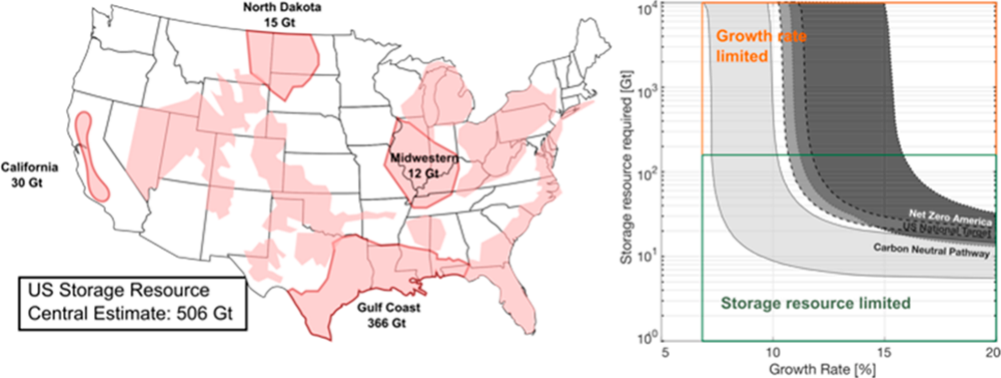

To progress decarbonization in the United States, numerous techno-economic models that project CO_2_ storage deployment at annual injection rates of 0.3–1.7 Gt year^–1^ by 2050 have been built. However, these models do not consider many geological, technical, or socio-economic factors that could impede the growth of geological storage resource use, and there is uncertainty about the feasibility of the resulting projections. Here, we evaluate storage scenarios across four major modeling efforts. We apply a growth modeling framework using logistic curves to analyze the feasibility of growth trajectories under constraints imposed by the associated storage resource availability. We show that storage resources are abundant, and resources of the Gulf Coast alone would be sufficient to meet national demand were it not for transport limitations. On the contrary, deployment trajectories require sustained average annual (exponential) growth at rates of >10% nationally for two of the three reports and between 3% and 20% regionally across four storage hubs projected in both reports with regional resolution. These rates are high relative to historical rates of growth in analogous large scale energy infrastructure in the United States. Projections for California appear to be particularly infeasible. Future modeling efforts should be constrained to more realistic deployment trajectories, which could be done with simple constraints from the type of modeling framework presented here.

## Abstract 2

Current projections of CO_2_ storage to reach net zero by 2050 in the United States are unconstrained. Growth modeling shows deployment trajectories of CO_2_ storage require annual growth rates of >10% nationally, which are high when compared to historical rates for other resources.

## Introduction

In most techno-economic model scenarios evaluating climate change mitigation, carbon capture and storage (CCS) is deployed at large scales, injecting CO_2_ underground at rates of gigatons per year by midcentury.^1^ The United States of America (USA) is one of the top emitters of CO_2_ and faces a significant challenge.^2^ In assessments of decarbonization in the USA, there are projected scenarios with up to 20 Gt of cumulative storage by 2050, with annual injection rates reaching nearly 2 Gt of CO_2_ year^–1^ by 2050.^3−6^ These amounts are large, with the envisioned scale for USA CO_2_ transport and storage being 2.4 times the current USA equivalent volume of oil production.^3^

There are some indications that this scale-up can be achieved.^2^ Currently, more than half (i.e., 14 of 26) of all operational, commercial, large scale CCS facilities are in the USA, with a combined capacity to capture nearly 20 million tonnes of CO_2_ per year.^7^ In 2020, due to the enhanced 45Q tax credit, 12 of the 17 new CCS facilities being developed globally are in the USA.^7^ The availability of storage resource is also a critical enabler.^8^ National volumetric-based evaluations of storage resources from the U.S. Geological Survey (USGS) and the U.S. Department of Energy (USDOE) estimate that there is 3000–6000 Gt of storage resource available, including deep saline aquifers, existing oil and gas fields, onshore, and state waters.^9,10^ Deep saline aquifers account for the majority (>97–99% of USDOE and USGS estimates) of the USA storage potential.^9,10^

At the same time, uncertainties remain around the rapid scale-up to injection at rates of gigatons per year.^11^ Storage resources may be less than the USDOE and USGS estimates^9,10^ when explicitly considering geophysical factors such as the injectivity of CO_2_ and reservoir pressure build-up, and constraints on subsurface plume migration due to the presence of faults or legacy wells that may provide leakage pathways.^12^ Teletzke et al.^12^ estimate a resource base of 506 Gt, which they refer to as the practicable storage resource base, when accounting for engineering and geological constraints. However, the techno-economic models used to identify CO_2_ storage demand in decarbonization pathways are predominantly constrained by the relative estimated price of technologies.^13−15^

As a result, gaps exist in the representation of storage resource consumption in these models.^16,17^ For instance, models underpinning the USA technology roadmaps consider an upper limit on the available storage resource and a maximum injection rate for CCS,^3^ but these limits are uncertain up to 2 orders of magnitude and do not usually result in limits to deployment. Consequently, the modeled deployment of CO_2_ from techno-economic models generally does not exhibit the types of growth trajectories observed in analogous technology.^13,18^ The techno-economic models are absent of a number of potential leading-order limitations to subsurface CO_2_ storage scale-up. These limitations could arise from the geophysical and engineering factors already mentioned, in addition to socio-economic factors, including time scales for achieving regulatory requirements, financing, and public acceptance.^19−23^

In this work, we use a logistic growth modeling framework to evaluate projected scale-up scenarios of subsurface CO_2_ storage in the USA. We analyze four major modeling efforts projecting climate change mitigation scenarios to 2050, the Net Zero America, Carbon Neutral Pathway, Long-Term Strategy, and Decarb America report. Logistic models are widely used for projections of growth pathways in analogous energy industries.^24,25^ This modeling framework is most frequently used in the analysis of natural resource consumption due to its parametrization linking rates of growth, largely driven by socio-economic factors, and the size of the natural resource base, driven by geophysical characteristics.^26−28^ This link is particularly useful in the analysis of CO_2_ storage deployment.^18^ Using this modeling framework, we evaluate the feasibility of projected CO_2_ storage scale-up at the national and regional scales in the USA.

## Materials and Methods

### 2.1 National Analysis Projections

We first analyze CCS scale-up nationwide for the USA. The United States’ commitment to tackling climate change has been reinstated following the election of the Biden–Harris administration. Alongside rejoining the Paris Agreement, a new nationally determined target has been announced, aiming at a 50–52% reduction in USA greenhouse emission from 2005 levels by 2030.^6^ Subsequently, several reports written by different organizations have been released, detailing decarbonization scenarios. We make use of three groups of national scenarios arising from these studies (Table 1).^3,4,6^

**Table 1:** National CO_2_ Storage Scenarios for the USA from Three Reports (Net Zero America, Carbon Neutral Pathways, and a Long-Term Strategy)^a^

report	scenario	storage rate demand (Gt of CO_2_ year^–1^)	cumulative storage demand (Gt)
Net Zero America	E+	0.9	10
	E–	1.5	17
	E+RE–	1.7	20
Carbon Neutral Pathways	central	0.316	4
	delayed electrification	0.38	5.5
	low land	0.68	5.5
	net negative	0.465	4.7
Long-Term Strategy	low	0.78	N/A
	medium	0.91	N/A
	high	1.04	N/A

a Each scenario includes a storage rate demand and an associated cumulative storage demand for 2050 unless indicated otherwise. The Long-Term Strategy did not provide associated cumulative storage projections; this is indicated by N/A in the table.

The first group of national scenarios comes from the Net Zero America study, a Princeton University-led, industry-funded academic research project that investigates possible technological pathways to net zero by midcentury for the USA.^3^ Within the Net Zero America report, six approaches to nationwide decarbonization have been outlined, including a reference scenario and a scenario excluding any subsurface sequestration of CO_2_ (100% renewable scenario). From this, three core scenarios with distinctly different levels of demand for CO_2_ storage are presented in this study: the E+ (high electrification) scenario storing 10 Gt of CO_2_ cumulatively with an annual injection rate of 0.9 Gt year^–1^ by 2050, the E– (less-high electrification) scenario with demands of 17 Gt of cumulative storage and an annual storage rate of 1.5 Gt year^–1^ by 2050, and E+RE– (constrained renewable) scenario stating 20 Gt of cumulative storage and an annual storage rate of 1.7 Gt year^–1^ by 2050^3^ (Table 1).

A second group of national scenarios comes from the Carbon Neutral Pathway report, an academic study funded by the United Nations Sustainable Development Solutions Network.^4^ A total of eight scenarios are described in the Carbon Neutral Pathway analysis, and in each scenario, a cumulative storage demand and an associated storage rate demand for 2050 are outlined. We analyze four of these scenarios with varied CO_2_ storage demands, labeled “central”, “delayed electrification”, “low land”, and “net negative”, with cumulative storage demands ranging from 4 to 5.5 Gt and storage rate demands between 0.3 and 0.7 Gt year^–1^ (ref (4) and Table 1).

A final group of national scenarios are derived from the “Long-Term Strategy of the United States”. This is a report in which the USA government outlines various decarbonization pathways^6^ that have been submitted to the United Nations Framework Convention on Climate Change (UNFCCC) under the Paris Agreement.^29^ To reach net-zero emissions by 2050, CO_2_ injection rates of 0.78–1.04 Gt yr^–1^ are proposed^6^ (Table 1).

The ramp-up of CO_2_ storage in these reports to some extent depends on the scale-up of CO_2_ capture across technologies, such as bioenergy with CCS (BECCS) and direct air capture (DAC). The scale-up of BECCS in turn is predominantly limited by the overall biomass supply. Across reports, similar biomass potential is represented within techno-economic models, which is derived from the USDOE 2016 Billion Ton Study.^30^ In the Net Zero America model, to avoid the high costs of biomass transportation given its low bulk density, biomass utilization must occur within 100 miles of its production.^3^ The Carbon Neutral Pathway assessment did not consider the CO_2_ capture resolved at the regional scale. Therefore, no details for biomass transportation were considered. The Long-Term Strategy report did not specify the methodology on how the constraint on biomass was applied to limit BECCS deployment.^6^ Overall, similar limitations for the ramp up of BECCS exist as with the representation of CO_2_ storage; there is significant uncertainty of the impact of regulation across states, political support, public acceptance, the intensification of energy crop production in competition with land for food and energy feedstock, and environmental impacts. These limitations are not in consideration by these models yet might inhibit the role of BECCS.^31^ Direct air capture deployment across all reports remains significantly limited, given its high cost and uncertainty in future deployment. However, given the focus of this study is geological CO_2_ storage irrespective of the CO_2_ source, assessments of the feasibility from the capture perspective are beyond the scope of this analysis.

The reports have varying representations of the storage resource base. The Carbon Neutral Pathway analysis did not include an upper limit for the storage resource base; a maximum annual injection rate of 1.2 Gt year^–1^ was used to constrain the deployment of CO_2_ storage.^4^ The Long-Term Strategy report did not specify any geological constraints used to model the deployment of CO_2_ storage.^6^ The most sophisticated consideration of the resource base came from the Net Zero America report that was based on the analysis of Teletzke et al.^12^ Teletzke et al.^12^ applied a series of technical and geological restrictions to the USGS’s theoretical estimates of storage resources (3000 Gt) over the same geographical distribution.^21^ They identified 506 Gt that could be practically exploited for storage of CO_2_ at the national level.^12^ In the Net Zero America report, the majority of storage took place in the Gulf Coast region,^3^ which contains >72% of the total estimated storage resource available in the USA (366 Gt).^12^ Moreover, the CO_2_ is stored in deep saline aquifers in the Net Zero America report, where they viewed a lack of commerciality in the use of a significant amount of CO_2_ storage with enhanced oil recovery.^3^

The USA has the longest record of injecting anthropogenic CO_2_ into the subsurface, albeit for enhanced oil recovery. The Terrel natural gas plant in southern Texas commenced in 1972 and began capturing CO_2_ through its natural gas stream, injecting the CO_2_ into a nearby oilfield for enhanced oil recovery.^33^ As of 2020, there are 13 operational projects in the USA injecting anthropogenic CO_2_ for storage, reaching an annual capture capacity of 21 Mt of CO_2_ year^–1^.^7^ According to databases maintained by the Global CCS institute^7^ and the International Oil and Gas Climate Initiative,^34^ 22 new CCS projects in the USA are planned, with operational start dates before 2030. At present, the overall CCS development in the USA since 2000 is commensurate with storing potentially 1 Gt of CO_2_ cumulatively by 2030. Note that actual storage rates have thus far been 19–30% less than capture capacity, but growth in storage rates and capture capacity is similar.^35^

### 2.2 Regional Analysis Projections

In addition to analyzing national storage deployment, we use two studies providing state-by-state technology portfolios, the Net Zero America study, and a report provided by the Decarb America research initiative^3,5^ (Table 2). For these studies, we apply our analysis at the regional scale.

**Table 2:** Regional CO_2_ Storage Scenarios for the USA from Two Reports (Net Zero America and Decarb America)^a^

report	storage rate demand (Mt of CO_2_ year^–1^)	storage hub
Net Zero America E+ scenario	47	North Dakota
	33	Midwestern
	769	Gulf Coast
	80	California
Decarb America high electrification	32	North Dakota
	265	Midwestern
	674	Gulf Coast
	63	California

a The aggregated storage rate demand is shown to be associated with a particular storage hub.

**Figure 1:**
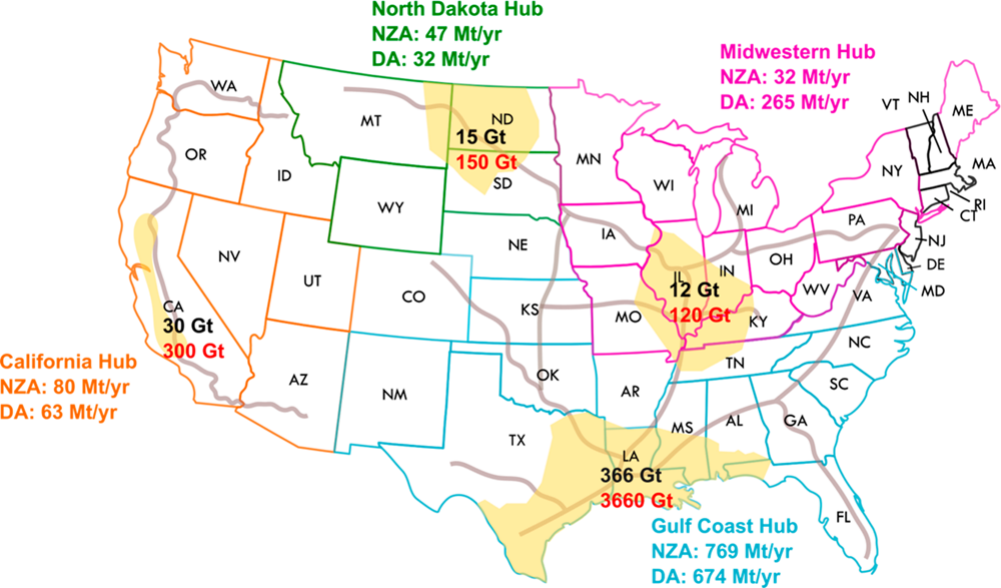
Map of the United States showing the regional central estimates (black text) and the maximum estimates (red text) of storage resource in the USA. All annual storage demands are for 2050. Pink, green, orange, and blue outlines denote the states included in the Midwestern, North Dakota, California, and Gulf Coast Hub, respectively. These states are determined on the basis of the connectivity of the pipeline (light brown lines). Yellow polygons indicate major storage resource locations analyzed by the USGS^22^ national assessment of geologic carbon storage resources.

In the Net Zero America study, state level CO_2_ transport infrastructure and storage systems were modeled for the E+ scenario. Figure 1 highlights the source-to-sink flows based on the modeled CO_2_ pipelines from the Net Zero America study (light brown lines in Figure 1). The pipeline system derived in the Net Zero America report is based on the annual flows of captured CO_2_ that are geographically distributed according to the geospatially located point sources in their model. Carbon dioxide transmission corridors in their report were created by hand to connect the largest number of CO_2_ sources with storage basins while minimizing the required transmission and spur pipelines.^3^ The allocation of CO_2_ capture to geospatially located point sources within each sector was designed in the Net Zero America analysis to be consistent with the national capture totals projected by its techno-economic assessment model.^3^

The second study we used for the regional analysis is a report created by the Decarb America research initiative, which documented state and regional storage demand.^5^ This is led by nonprofit organisations and policy think tanks, including the Clean Air Task Force. They have looked at various technology pathways for the USA to reach net-zero greenhouse gas emissions by 2050.

Six priority regions were identified by the Net Zero America report for CO_2_ storage site characterization, linked by the pipeline network. From this, we select those with the most abundant storage resource available based on the estimates of practicable storage resources from Teletzke et al.^3,23^ These are California (30 Gt; bold black text in Figure 1), the Gulf coast (366 Gt), North Dakota (15 Gt), and five Midwestern states (Illinois, Indiana, Kentucky, Tennessee, and Missouri; 12 Gt). We also indicate a maximum upper bound on the regional geological availability for each hub (blue text in Figure 1). Currently announced plans for CCS in each hub are commensurate with storing 12 Mt of CO_2_ (California), 455 Mt of CO_2_ (North Dakota), 360 Mt of CO_2_ (Gulf Coast), and 122 Mt of CO_2_ (Midwestern) by 2030.^7,34^

For comparison with the Net Zero America analysis, we aggregate state level storage demands from the Decarb America report into the four regional storage hubs identified from the Net Zero America study. For the Net Zero America study, the regional storage demands are 80 Mt year^–1^ by 2050 (California), 769 Mt year^–1^ by 2050 (Gulf Coast), 47 Mt year^–1^ by 2050 (North Dakota), and 32 Mt year^–1^ by 2050 (Midwestern). The Decarb America regional storage demands are 63 Mt year^–1^ by 2050 (California), 674 Mt year^–1^ by 2050 (Gulf Coast), 32 Mt year^–1^ by 2050 (North Dakota), and 265 Mt year^–1^ by 2050 (Midwestern).

### 2.3 Growth Trajectories Using the Logistic Modeling Framework

The logistic model is one of several sigmoidal curves used to describe patterns of growth in natural resource consumption, including the cumulative normal curve. It has been widely employed in various sectors across energy and technology domains.^24−28,36−45^ Comparisons applying different sigmoid models show that so long as the curvature of the model is symmetric, the outcomes remain qualitatively similar.^24,27^ In the context of subsurface resources such as coal, these models are differentiated by their ability to predict particular curve features but the variation of outcome is within the range of uncertainty arising from the subsurface.^27,46,47^ The logistic curve is characterized by an initial phase that is close to exponential (constant annual growth). This is often taken to represent a stage of commerciality when a broad market-driven expansion of resource consumption begins.^37^ In latter stages of the modeled trajectories, growth slows, reaches a peak, and then declines. These phases represent the direct and indirect impingement on growth from geological constraints arising from the decreasing availability of high-quality reservoirs.^19^

This modeling framework was applied in the context of CCS to evaluate the global aggregate storage resource requirements for CCS scale-up by the end of the century.^18^ The use of the logistic model allows a representation of the connection among geophysical factors, the physical quantity of subsurface geology suitable for CO_2_ storage, and techno-economic dimensions (regulations, financing, latencies in project development, and public acceptance) that will underpin growth trajectories of deployment. In the limiting case in which geological resources are sufficiently abundant, trajectories will fall along the early part of the logistic curve and will be approximately the same as an exponential model.

The uncertainty around the geological resource base at very large scales of deployment is one reason for the use of growth models. A large set of geological parameters that vary widely across geological and geographical settings governs the effectiveness of CO_2_ storage.^48^ Detailed site level resource assessments are needed to define known quantities of a storage resource.^8^ A positive final investment decision is also required to qualify the resource as commercially recoverable.^8^ Given the complexities involved in project development and an absence of empirical data from an emerging CCS market, geologically based resource assessments have irreducible uncertainties that range over 1–2 orders of magnitude for saline aquifers.^18,48^ The growth modeling framework allows for a representation of the inhibiting impact of a potentially limiting resource base on growth.

The logistic modeling framework is also useful for quantitively identifying key features arising from the combined consideration of the geographical distribution of the resource base with storage resource use. Zhang et al.^19^ demonstrate this in the case of European climate change mitigation plans. Most plans could have been identified as ambitious from a growth rate perspective with a simple exponential growth model. However, the logistic modeling framework further allowed the quantitative identification of the storage resource of the North Sea as sufficient to serve as a regional hub for CO_2_ from continental Europe.

There are limits of applicability of the modeling framework when the resource base becomes small.^19^ In our analysis for the USA, we avoid using the model to monitor storage demand projections that are earlier than 2050. Similarly, our geographical consideration aggregates storage resource and demand from multiple states for several of the identified hubs so that at least scores of sites would be required to meet demand for a given region.

### 2.4 Definition and Constraints of the Logistic Growth Model

**Figure 2:**
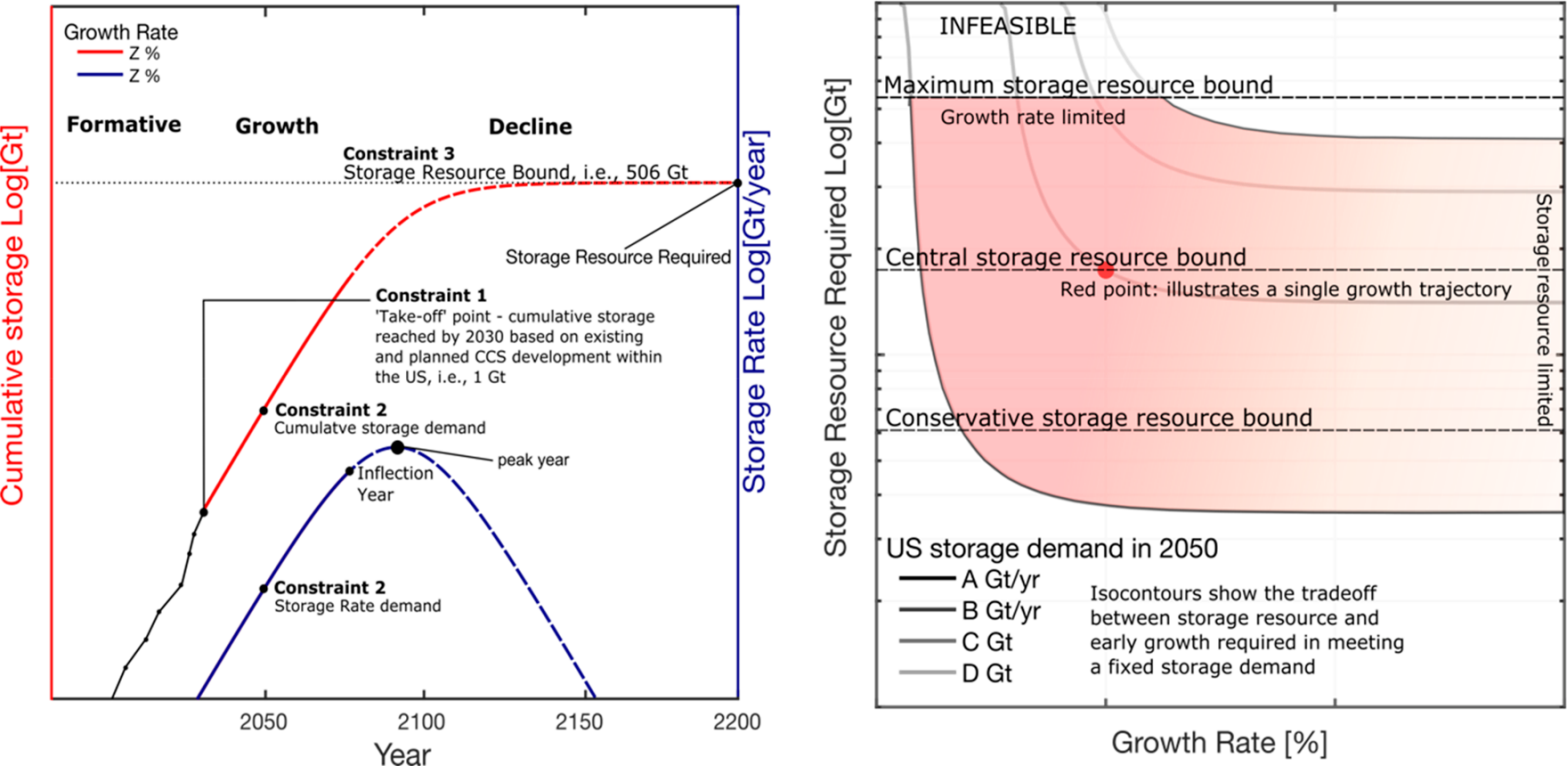
Schematic plot illustrating the constraints and key features of the logistic modeling framework using an exemplary growth trajectory of *Z*% (left). Equation 1 describes the cumulative storage of CO_2_ (red), and equation 2 describes the annual CO_2_ injection rate (blue). The black line joined by dots represents the cumulative storage from existing and planned CCS facilities. Exemplary plot illustrating the trade-off relationship between storage resource requirement growth rate bound by three levels of geological constraints (right). Above the maximum geological constraint, modeled scenarios are considered infeasible. The gradational color change indicates an evolution of the storage demand from growth rate limited (pink) to storage resource limited (white). Note that the plots are for illustrative purposes. Numbers are not included; however, the vertical axes are logarithmic, and the horizontal axes are linear.

To constrain the starting point of the growth trajectories, we make use of the stated capture capacity for projects that are listed in the 2020 Global status report by the Global CCS Institute^7^ and the database by the Oil and Gas Climate Initiative^34^ to estimate cumulative storage projected to 2030. These projects include both operational and planned CCS activities. Cumulative storage by 2030 is therefore what we assume to mark the takeoff point for CCS deployment (Figure 2). For national scenarios, cumulative storage identified for 2030 is rounded up to 1 Gt. For the individual regional hubs, these are 0.01 Gt by 2030 for California, 0.46 Gt by 2030 for North Dakota, 0.12 Gt by 2030 for Midwestern, and 0.36 Gt by 2030 for Gulf Coast.

We use a three-parameter, symmetric logistic growth model given in eqs 1 and 2 to describe the modeled cumulative storage, *P*(*t*) (gigatons of CO_2_), and storage rate, *Q*(*t*) (gigatons of CO_2_ per year), of CO_2_ storage at time *t* (years), respectively. The growth curve is characterized by an initial near-exponential growth at a rate characterized by the parameter *r* (inverse years), which hereafter is termed the growth rate. Upon approaching the peak year, *t*_p_ (years), growth declines until the storage resource base, *C* (gigatons), is reached.
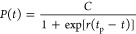
1
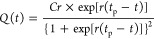
2We used the first inflection point in the rate time series as the end of the initial exponential trend. This occurs in year *t*_n_ after approximately 20% of the resource is consumed, with the time given by
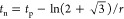
3The cumulative and rate trajectories to achieve CO_2_ storage demands for 2050 are determined through solutions to eqs 1 and 2, which are found numerically (left graph of Figure 2). Each storage demand or target outlined in the Net Zero America, Carbon Neutral Pathway, Long-term Strategy, and Decarb America reports^3−6^ is used as a second constraint in our model. These trajectories are not predictive, and there are limitations within logistic models to accurately describe declines in resource consumption.^35^ To emphasize that we use the model to identify feasible trajectories for the initial growth phase, we show dashed lines for the curve beyond the inflection year (Figure 2). Once we have identified individual trajectories of interest, we iterate computationally, finding every combination of the growth rate and storage resource requirement that meets a fixed CO_2_ storage demand.

We reformulate this information into graphs showing the trade-off inherent in the model between growth rate and storage resource requirements (right graph of Figure 2). Teletzke et al.^12^ applied geological and technical constraints to an estimate by the USGS^9^ to identify a resource base that could be practically accessed, and we use this as the central scenario on the trade-off graph (Figure 2). We analyze the sensitivity of the storage resource base on growth by exploring resource estimates ranging over 2 orders of magnitude, the range of uncertainty when multiple storage resource assessments have been performed on the same region.^18^ Our maximum upper bound is an order of magnitude higher than the estimates of Teletzke et al.^12^ This leads to an upper bound similar to the static estimates over the same geographic region.^9,10^

We use as a conservative lower bound 10% of the estimate from Teletzke et al.^12^ in both national and regional analyses. This lower bound results in a very small resource base, 50 Gt nationally and as little as 3 Gt in California, sufficient to support only a small number of projects. We include these values as an end-member to illustrate the impact that a limited resource base could have on achieving deployment targets. However, with such a small resource base, many factors, including a lack of business confidence, may preclude the development of logistic growth. In the national models, we also evaluate the potential for the Gulf Coast to act as a national hub, serving the entire national demand for CO_2_ storage, by constraining the growth trajectories with a storage resource total of 366 Gt. This ignores likely limitations arising from the necessary transport infrastructure for such a scenario but illustrates the significance of the Gulf Coast as a major potential storage resource for the USA.

The location of the proposed storage demand on the resource growth rate graph is an indication of the feasibility of the proposed scenario. Scenarios meet the minimum standard of feasibility if they are within the bounding limits of the graph: the maximum estimate of available storage resources and an initial exponential growth rate *r* < 20% (colored region on the right graph of Figure 2, eqs 1 and 2). At extreme ends of the curves, scenarios can be considered to exhibit low feasibility, as they are limited by either storage resource availability or the requirement of a historically high sustained rate of growth.

## 3 Results and Discussion

### 3.1 Net Zero America National Scenarios

**Figure 3:**
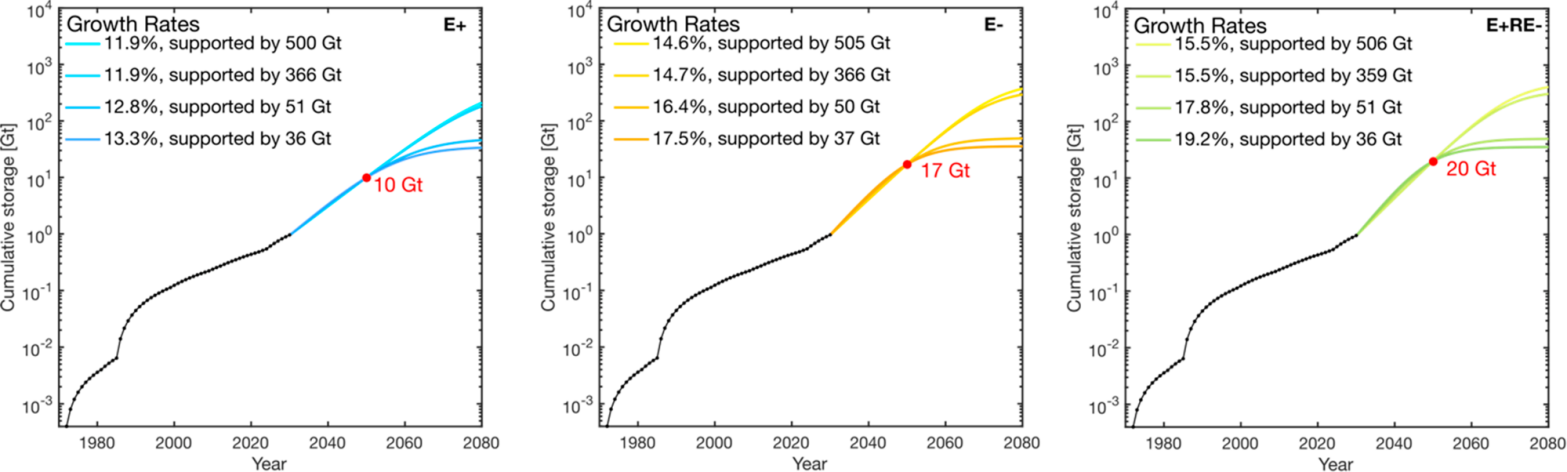
Cumulative CO_2_ storage as a function of time for three Net Zero America scenarios to meet 2050 cumulative storage demands (10, 17, and 20 Gt; red points). The cumulative CO_2_ injection based on existing and planned CCS facilities is indicated by black markers. We compare the range of growth rates required to meet storage demands at four storage resource bounds of 506 Gt (central estimate of the USA), 366 Gt (central estimate of the Gulf Coast), and 10% of each estimate. Model parameters are summarized in Table 3.

We first discuss short-term cumulative storage and storage rate trajectories over a range of rates from 11% to 20%, from 2030 onward to meet published CO_2_ storage demands of the E+, E–, and E+RE– scenarios from the Net Zero America report in Figure 3, considering the central storage resource estimate of 506 Gt. The lowest growth rate required is 11.9% to achieve the cumulative storage projections of 10 Gt in the E+ scenario (cyan curve in Figure 3). For the more ambitious scenarios of E– (17 Gt) and E+RE– (20 Gt), the required growth rates are 14.2% and 15.5%, respectively (light yellow and light green curves, respectively, in Figure 3).

The cumulative storage demand can still be met when considering scenarios with a more restricted resource base. The storage demands can be supported by the storage resource estimated to be available in the Gulf Coast alone (366 Gt), with a small increase of <0.1% for each growth rate identified. When the USA storage resource base is constrained to only 10% of the current estimates, the storage demand can still be met, but much higher initial growth rates are required, of at least 12% and ≤20% (Figure 3). This is because the limited resource base results in a slowdown of deployment below the exponential growth rate before the targets are achieved.

**Figure 4:**
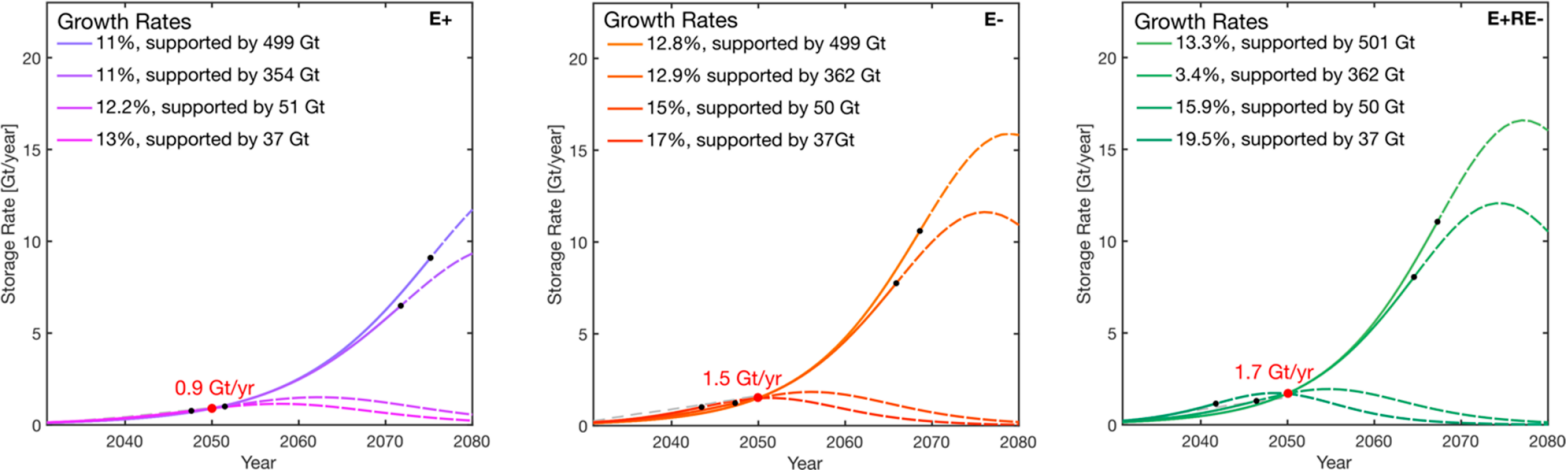
Plot showing the CO_2_ storage rate as a function of time for Net Zero America scenarios to meet storage rate demands for 2050 (0.9, 1.5, and 1.7 Gt year^–1^; red points). The legend shows the logistic curve growth rate from 2030 onward and the necessary storage resource required to support that growth at various storage resource bounds. The gray dashed lines illustrate the modeled pathway in the Net Zero America report for each scenario. Model parameters are summarized in Table 3.

If storage rate, rather than cumulative storage, is the target, growth rates needed to meet the demand of E+ (0.9 Gt year^–1^), E– (1.5 Gt year^–1^), and E+RE– (1.7 Gt year^–1^) for 2050 are lower, with the exception of the E+RE– scenario [19.5% of growth supported by 10% of the storage resources available in the Gulf Coast (Figure 4)].

The identified growth rates that can meet both the cumulative and annual storage demands for these three scenarios are within a similar range, between 13% and 18%. A summary of the results from Net Zero America scenarios is provided in Table 3.

**Table 3:** Summary of Modeled Growth Trajectories and Storage Resource Requirements That Correspond to Colored Lines in Figures 3 and 4

scenario	growth rate (%)	storage resource required (Gt)	demand achieved (cumulative or rate)
E+	11.9	500	10 Gt
	11.9	366	10 Gt
	12.8	51	10 Gt
	13.3	36	10 Gt
E–	14.6	505	17 Gt
	14.7	366	17 Gt
	16.4	50	17 Gt
	17.5	37	17 Gt
E+RE–	15.5	506	20 Gt
	15.5	359	20 Gt
	17.8	51	20 Gt
	19.2	36	20 Gt
E+	11	499	0.9 Gt year^–1^
	11	354	0.9 Gt year^–1^
	12.2	51	0.9 Gt year^–1^
	13	37	0.9 Gt year^–1^
E–	12.8	499	1.5 Gt year^–1^
	12.9	362	1.5 Gt year^–1^
	15	50	1.5 Gt year^–1^
	17	37	1.5 Gt year^–1^
E+RE–	13.3	501	1.7 Gt year^–1^
	13.4	362	1.7 Gt year^–1^
	15.9	50	1.7 Gt year^–1^
	19.5	37	1.7 Gt year^–1^

Meeting Combined Storage Demands			
scenario	growth rate (%)	storage resource required (Gt)	demand achieved (cumulative and rate)
E+	13.6	30	10 Gt and 0.9 Gt year^–1^
E–	17.7	36	17 Gt and 1.5 Gt year^–1^
E+RE–	18.3	38	20 Gt and 1.7 Gt year^–1^

### 3.2 Carbon Neutral Pathway National Scenarios

**Figure 5:**
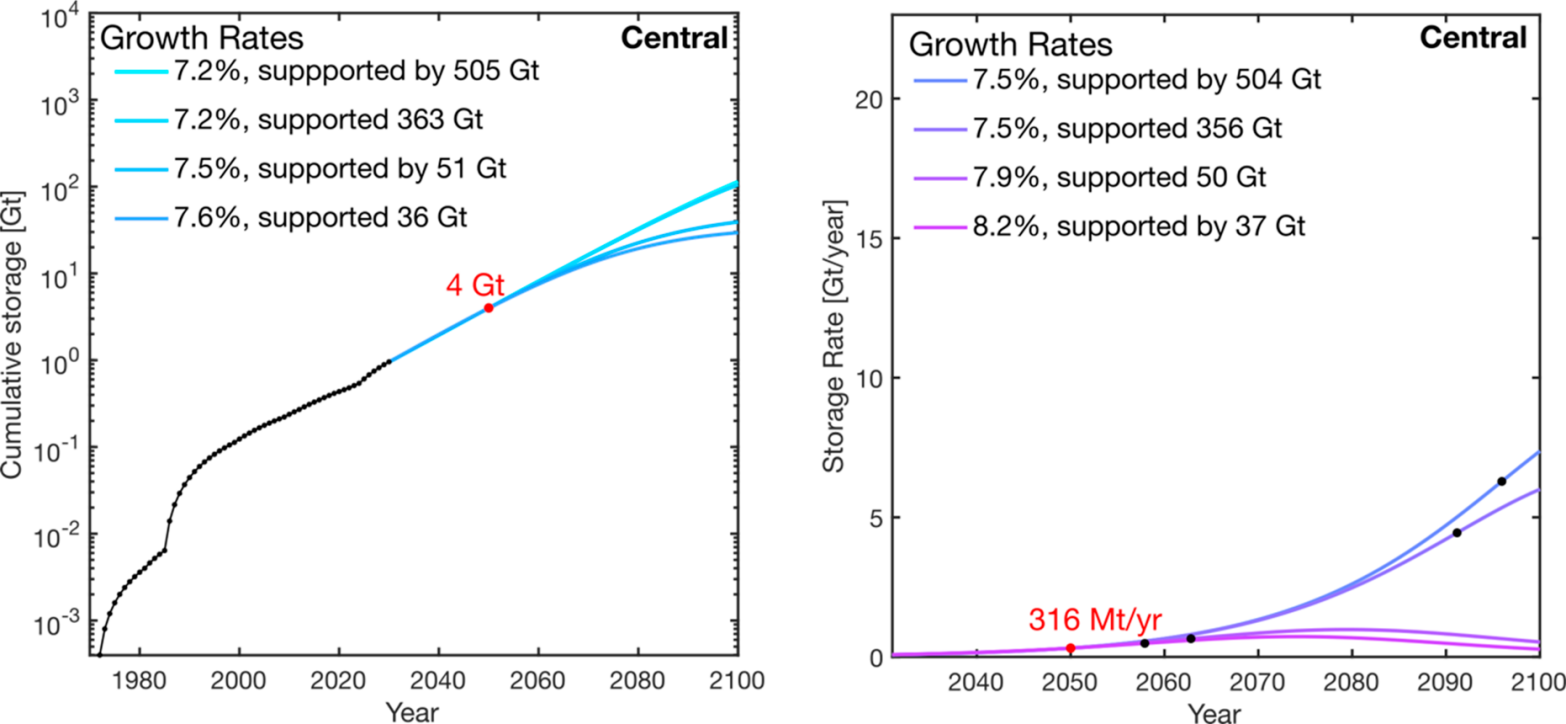
CO_2_ cumulative storage for the central scenario from the Carbon Neutral Pathway report (left). Plot of the corresponding CO_2_ storage rate as a function of time for the central scenario to meet an associated storage demand of 316 Mt year^–1^ by 2050 (right). Within each plot, we compare the necessary growth rate required to meet the modeled storage demand for 2050 constrained at various storage resource bounds. Model parameters are summarized in Table 4.

Cumulative storage demands ranging from 4 to 6 Gt, and storage rate demands ranging from 0.3 to 0.7 Gt year^–1^ by 2050, are outlined in the Carbon Neutral Pathway analysis^4^ (Table 1). We show growth rates between 7% and 10% are required from 2030 onward, depending on various storage resource constraints to meet the demands of the central scenario (4 Gt by 2050), the delayed electrification and low-land scenario (5.5 Gt by 2050), and the net negative scenario (4.7 Gt by 2050). Similarly, to meet storage rate demands of 316–680 Mt of CO_2_ year^–1^, a range of growth rates of 7–11% are needed subject to various storage resource constraints (additional figures are provided in the Supporting Information). Figure 5 shows an illustrative plot of cumulative storage and storage rate trajectories modeled for the central scenario. Only the delayed electrification scenario has a trajectory (growth rate of 10%) that meets both the cumulative and annual storage demand. Our modeling framework could not fit growth trajectories constrained by both modeled annual storage and cumulative demands within the scenarios of central, low land, and net negative. A summary of the outcomes is provided in Table 4.

**Table 4:** Growth Model Parameters of the Central Scenario from the Carbon Neutral Pathway Report Corresponding to the Lines in Figure 5^a^

scenario	growth rate (%)	storage resource required (Gt)	demand achieved (cumulative or rate)
central, cumulative	7.2	505	4 Gt
	7.2	363	4 Gt
	7.5	51	4 Gt
	7.6	36	4 Gt
central, storage rate	7.5	504	316 Mt year^–1^
	7.5	356	316 Mt year^–1^
	7.9	50	316 Mt year^–1^
	8.2	37	316 Mt year^–1^

Meeting Combined Storage Demands			
scenario	growth rate (%)	storage resource required (Gt)	demand achieved (cumulative and rate)
central	N/A	N/A	4 Gt and 316 Mt year^–1^
delayed electrification	10.5	18	5.5 Gt and 380 Mt year^–1^
low land	N/A	N/A	5.5 Gt and 680 Mt year^–1^
net negative	N/A	N/A	4.7 Gt and 465 Mt year^–1^

a N/A denotes scenarios in which the logistic model could not fit the combined storage constraints.

### 3.3 Long-Term Strategy for National Scenarios

**Figure 6:**
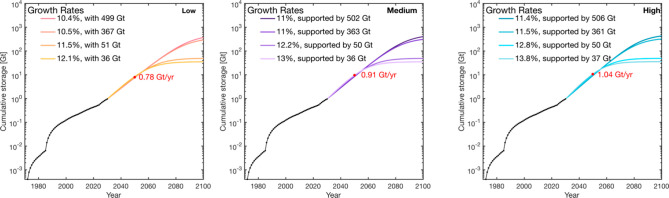
Cumulative CO_2_ storage plot as a function of time for Long-Term Strategy scenarios to meet 2050 storage rate projections (0.78, 0.91, and 1.04 Gt year^–1^; red points). Cumulative CO_2_ injection based on existing and planned CCS facilities is indicated by black markers. We compare the range of growth rates required to meet storage demands at four storage resource bounds of 506 Gt (central estimate of the USA), 366 Gt (central estimate of the Gulf Coast), and 10% of each estimate. Model parameters are summarized in Table 5.

Three storage rate scenarios ranging from 0.78–1.04 Gt of CO_2_ year^–1^ are projected as part of the Long-Term Strategy of the USA to reach net-zero greenhouse gas emission by 2050.^6^ We show storage resource-constrained, growth rates between 10.4% and 13.8% are required to meet these projections (Figure 6). Within each scenario, to meet a given storage projection, higher growth rates are required to compensate for the constrained storage resource available. Furthermore, across scenarios, given the same storage resource constraint, higher storage rate projections require that a higher growth rate be achieved from 2030. A summary of the outcomes is provided in Table 5.

**Table 5:** Growth Model Parameters of Three Storage Rate Scenarios from the Long-Term Strategy Report Corresponding to the Lines in Figure 6

scenario	growth rate (%)	storage resource required (Gt)	projection achieved (Gt year^–1^)
low	10.4	499	0.78
	10.5	367	0.78
	11.5	51	0.78
	12.1	36	0.78
medium	10.97	502	0.91
	11	363	0.91
	12.2	50	0.91
	13	36	0.91
high	11.4	506	1.04
	11.5	361	1.04
	12.8	50	1.04
	13.8	37	1.04

### 3.4 Comparison of the National Scenarios: Net Zero America, Carbon Neutral Pathway, and Long-Term Strategy

**Figure 7:**
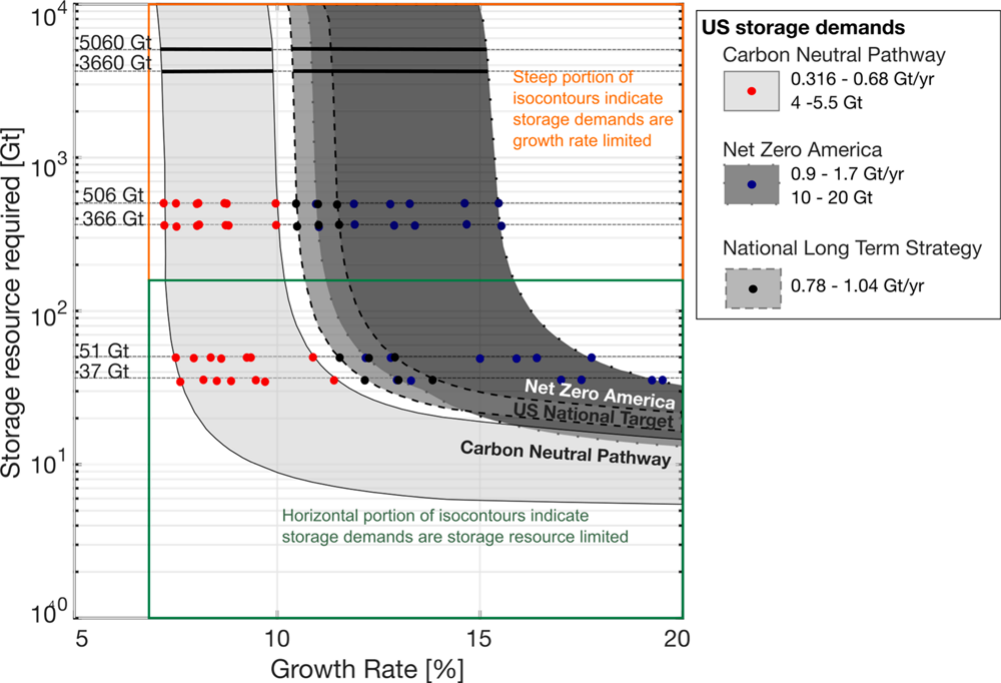
Trade-off between storage resource requirement and growth rates for 2050 USA storage demands and scenarios illustrated with three ranges of isocontour bands representing scenarios from the different reports. The points are colored depending on the report from which they originated and correspond to growth rates subject to various storage resource constraints that we have investigated, including 506 Gt (central estimate for the USA), 366 Gt (central estimate for the Gulf Coast), and 1 order of magnitude higher and lower.

A comparison of national scenarios from the Net Zero America, Carbon Neutral Pathway, and the Long-Term strategy reports is shown in the trade-off graph in Figure 7. The three gray regions indicate the range of isocontours with combinations of growth and storage resource requirements that meet 2050 storage projections. The points indicate growth scenarios modeled in Figures 3–6 that are bounded by the central storage resource estimate for the USA (506 Gt) and the Gulf Coast (366 Gt).^12^ The impact of uncertainty in the resource base is illustrated by horizontal lines an order of magnitude less and greater than the central estimates (37–3660 Gt for the Gulf Coast and 51–5050 Gt for the USA).

From the perspective of storage resource availability, all projections from the reports are feasible. The minimum storage resource base required to accommodate any potential scenario is 5 Gt, with at least 37 Gt of storage resources being required for all CO_2_ storage projections to be viable. This is outside the uncertainty range of the current central storage resource base estimates in the USA.

On the contrary, scenarios in the Net Zero America report and the National Long-Term Strategy have CO_2_ storage demands that require sustained growth of >10%, and ≤16%. This is comparable with the range of growth trajectories identified to meet European CO_2_ injection projections.^19^ These scenarios are growth rate limited; the growth rate requirements are driven by 2050 storage scenarios and are not limited by the storage resource available. Increasing the storage resource base from the central case to the maximum bounds of 3660 and 5060 Gt has a marginal impact (∼0.1% change) on decreasing the growth rate requirement. This is illustrated by the solid bold black lines in Figure 7. In contrast, scenarios in the Carbon Neutral Pathway report require more modest growth of 7.2–8.2%.

Impacts of storage resource limitations emerge for all of these scenarios only if the available storage resource base is ≤10% of the central estimates, that is the practicable estimates of Teletzke et al.^12^ In such a case, to achieve a given storage demand, a higher growth rate is required to compensate for the geological limitations; this is more apparent for storage demands from the Net Zero America report. In contrast, because of the lower storage demand in the Carbon Neutral Pathway report, growth rate requirements remain almost unchanged (<0.1% difference in growth rate) when the storage resource is limited to 10% of current central estimates.

### 3.5 Comparison of Regional Scenarios: Net Zero America and Decarb America Hubs

**Figure 8:**
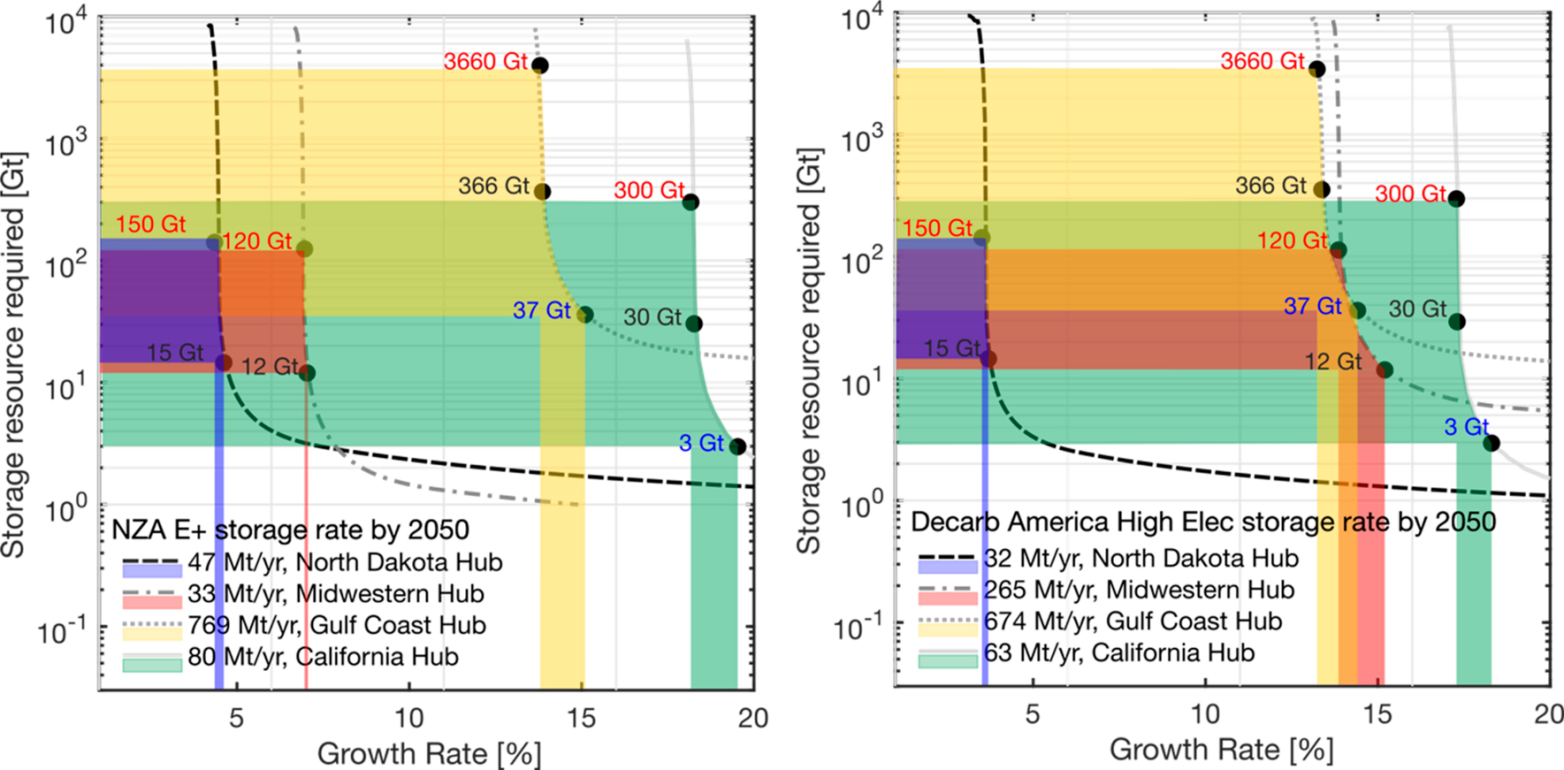
Trade-off between the storage resource requirement and growth rates for four regional hubs meeting modeled demands analyzed in the E+ scenario of the Net Zero America report for 2050 (left). Trade-off between the storage resource requirement and growth rates for four regional hubs meeting modeled demands in the high-electrification (E+) scenario from the Decarb America report (right). Colored points represent growth rates subject to various storage resource bounds. The maximum estimates are given in red text, the central estimates in black text, and the conservative estimates in blue text (see section 2.1). Colored regions show how the range of variation within storage resource bounds on the *y* axis results in varying required growth rates on the *x* axis.

The regional scenarios for Net Zero America and Decarb America are compared in Figure 8. The points in Figure 8 represent growth rates that are dependent on either the maximum storage resource estimates (red text in Figure 8), the central storage resource estimates (black text in Figure 8), or the conservative resource estimates (blue text in Figure 8). The range of growth rate requirement from 2030 onward is between 3% and 20%. The colored area indicates the range of growth rate variation resulting in the variation of storage resource considered.

The largest difference between the reports arises from the significantly higher storage rate demand (265 Mt year^–1^) in the Midwestern hub in the Decarb America scenario, 8 times greater than the equivalent hub from the Net Zero America report (33 Mt year^–1^). As a result, the growth rate required to meet the demand is more than five percentage points higher in the Decarb America Scenario (15% and 7% for the Decarb America and Net Zero America reports, respectively, under the central storage resource constraint).

In both reports, the implied scale-up of storage deployment in California is very high. California has no existing subsurface CO_2_ storage operations, and the first project is planned to be in operation by 2025. As a result, California must reach an annual injection rate of 63 80 Mt year^–1^ within a five-year window of this project starting to reach projections. Thus, the required scale-up is demanding. On the other end of the scale, in both the Net Zero American and Decarb America reports, the storage demand of the North Dakota region can be achieved with as little as 3% growth. This is evidently more plausible than the scenarios identified for the California hub.

All regional storage rate demands are considered feasible from the perspective of the available storage resources. The minimum storage resource base required for all storage rate demands to be viable is within the variation of the storage resource estimates.

### 3.6 Implications

The rates of growth required to meet projections are sensitive to project development prior to 2030 and, to a lesser extent, uncertainty in the storage resource base. Reviews of CCS pilot and demonstration projects from the past three decades have shown a significant number of projects end prior to operational start.^49,50^ Additionally, there is a discrepancy of 10–20% between the stated capture capacity and estimated storage amounts for CCS projects that do reach operation.^35^ Both issues suggest that actual growth in deployment to meet projections will be even higher than that estimated herein. As discussed by Zhang et al.^19^ and Zahasky and Krevor,^18^ a decreased storage resource results in an earlier slowing of development, requiring faster initial project growth to meet targets. However, the impact of considering uncertainty in storage resources on national and regional scenarios in Figures 7 and 8 is small, until estimated storage resources are reduced by ≥90% from the central estimates. Projected deployment is thus within feasible bounds from the perspective of storage resource estimates for the USA, even when considering the wide-ranging uncertainty in the resource base.

At the same time, double digit rates of growth must be sustained until 2050, and higher rates of project realization are needed over the coming decade, to meet projections from the USA government Long-Term Strategy and the Net Zero America report. The hydrocarbon industry provides one industrial analogue for evaluating the feasibility of growth trajectories for CO_2_ storage. National oil production in the USA achieved a sustained growth of 5% between 1926 and 1946.^52^ Regionally, offshore oil production of crude oil in the Gulf of Mexico sustained annual exponential growth of merely 3% between 1991 and 2011.^53^ In Europe, historical oil production on the U.K. continental shelf sustained an aggressive growth rate of 120% over a 10-year period from 1974 before growth declined. In contrast, the Norwegian sector of the North Sea, and the Norwegian Sea and Barents Sea, collectively achieved a growth rate of 35% over a 20-year period commencing from 1974.^54^ The rates for the U.K. and Norwegian North Sea are extraordinary in terms of their combined magnitude and duration, illustrating the impact that a mature industry could have on the acceleration of development in new areas, although distinct, regional historical oil production provides a snapshot of the market conditions that might be required to drive deployment of CCS to meet published storage scenarios of CO_2_ in the USA.

This analysis supports the prospect of the Gulf Coast serving as a major storage hub. One analysis used the historical construction of oil and gas wells in the Gulf of Mexico as a proxy for a pathway to scale-up of CO_2_ storage.^51^ This demonstrated that the scale of engineering required for gigaton scale injection rates by midcentury had precedent.^51^ A single “Gulf of Mexico” equivalent development for CO_2_ storage based on historical well construction will be able to inject 7 times the most ambitious scenario considered in this analysis in 2050 (1.7 Gt year^–1^). Within the modeled Net Zero America report, the Gulf Coast alone provides 75% of the annual storage capacity.^3^ Additionally, storage resources in North Dakota, the Midwest, and California are useful as storage hubs for local sources. However, as discussed above for California, there are challenging short-term growth requirements to meet the proposed 2050 storage rate projections.

Challenges remain with achieving the scale-up projected in these reports. The required pipeline network in the Net Zero America scenarios is potentially larger than the existing oil and gas pipe system.^3^ The urgency of CCS scale-up and the role of the federal government in leading the steep delivery of CO_2_ reduction are recognized by USA policy makers. Actions to implement policy and regulatory packages to achieve near-term and long-term goals are underway according to the Long-Term Strategy report released by the USA government,^6^ but the policy landscape is still inadequate for the envisioned storage demand in 2050. Real-world deployment has consistently fallen short of the expectations set by projections of the types presented in the reports studied herein.^55−61^ Our results identify incentives for growth, and not the geological resource base, as the key barrier to deployment in meeting published climate change mitigation scenarios in the USA. At the same time, our results also suggest that future mitigation modeling should include some scale-up trajectories for subsurface CO_2_ storage that are constrained by growth with historical precedent in the energy industry.

## Supporting Information Available

Additional results, including figures and a table for the Carbon Neutral Pathway scenarios (PDF)
Additional raw data of current capture capacity from existing and planned CCS projects in the USA (XLSX)

## Notes

Additional MATLAB scripts for the generation of logistic analysis available at https://github.com/flowrazhang/USCO2storage.

## Abbreviations

CCS: carbon capture and storage
CO_2_: carbon dioxide
UNFCCC: United Nations Framework Convention on Climate Change
USA: United States of America
USDOE: United States Department of Energy
