# Supplementary material for: Feasibility of Carbon Dioxide Storage Resource Use within Climate Change Mitigation Scenarios for the United States
Source: Environ Sci Technol. 2023 Sep 26;57(40):14938–49. doi: 10.1021/acs.est.3c00790 (PMC10569028; doi:10.1021/acs.est.3c00790)
Supplement: Supplementary file 1 — es3c00790_si_001.pdf [file es3c00790_si_001.pdf]

# The feasibility of carbon dioxide storage resource use within climate change mitigation scenarios for the USA

Yuting Zhang[a*], Christoper Jackson[a], Nihal Darraj[a], and Samuel Krevor[a]

[a] Department of Earth Science and Engineering, Imperial College London, Exhibition Road, London, SW7 2BX, UK

*Email: yuting.zhang16@imperial.ac.uk

## Supporting Information

Summary: this document includes the additional figures modelled for the Carbon Neutral Pathway scenarios. Model parameters are summarised in Table 1.

## Supporting Information

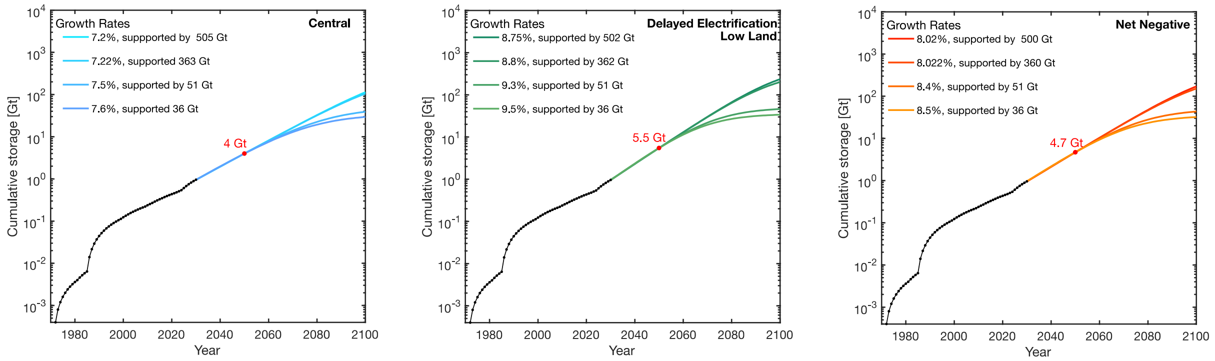


*Figure 1: Cumulative $CO_2$ storage plot as a function of time for Carbon Neutral Pathway scenarios to meet 2050 cumulative storage demands (4 Gt; 5.5 Gt; 4.7 Gt; red points). Cumulative $CO_2$ injection based on existing and planned CCS facilities is indicated by black markers. We compare the range of growth rates required to meet storage demands at four storage resource bounds of 506 Gt (conservative estimate of the US), 366 Gt (central estimate of the Gulf Coast), and 10% of each estimate. Model parameters are summarised in Table 1.*

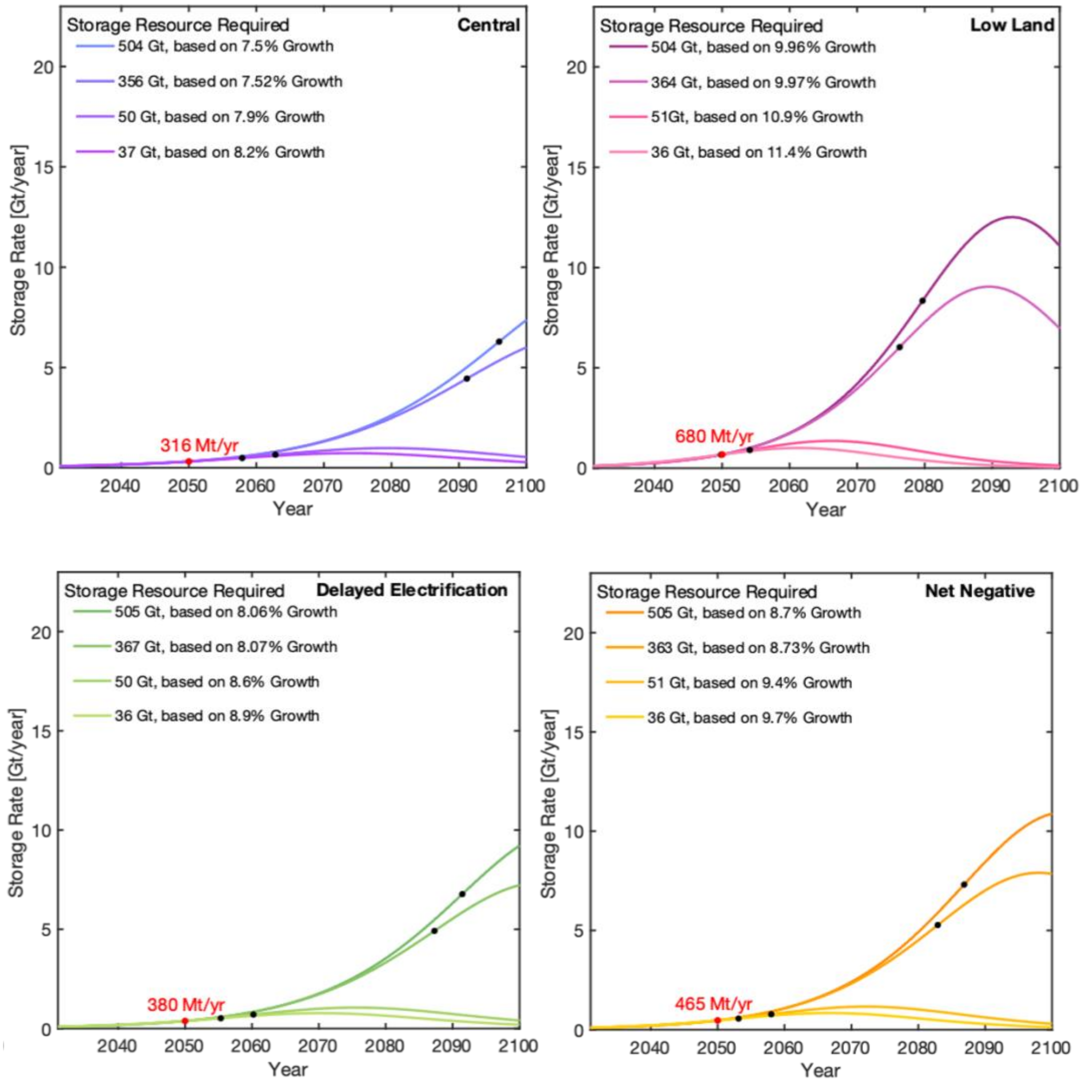


*Figure 2: Plot of corresponding $CO_2$ storage rate as a function of time to meet 2050 annual storage demands (316 Mt $yr^{-1}$; 680 Mt $yr^{-1}$; 380 Mt $yr^{-1}$; 465 Mt $yr^{-1}$; red points). Within each plot, we compare the necessary growth rate required to meet the modelled storage demand for 2050 constrained at various storage resource bounds. Model parameters are summarised in Table 1.*

*Table 1: Growth model parameters of scenarios from the Carbon Neutral Pathway report corresponding to lines in Fig.1 and 2.*

| *Scenario* | *Growth rate [%]* | *Storage resource required [Gt]* | *Demand achieved* |
|---|---|---|---|
| *Central - cumulative* | 7.2 | 505 | 4 Gt |
| | 7.2 | 363 | 4 Gt |
| | 7.5 | 51 | 4 Gt |
| | 7.6 | 36 | 4 Gt |
| *Central – storage rate* | 7.5 | 504 | 316 Mt $yr^{-1}$ |
| | 7.5 | 356 | 316 Mt $yr^{-1}$ |
| | 7.9 | 50 | 316 Mt $yr^{-1}$ |
| | 8.2 | 37 | 316 Mt $yr^{-1}$ |
| *Low land & Delayed Electrification - cumulative* | 8.8 | 502 | 5.5 Gt |
| | 8.8 | 362 | 5.5 Gt |
| | 9.3 | 51 | 5.5 Gt |
| | 9.5 | 36 | 5.5 Gt |
| *Low land – storage rate* | 10.0 | 504 | 680 Mt $yr^{-1}$ |
| | 10.0 | 464 | 680 Mt $yr^{-1}$ |
| | 10.9 | 51 | 680 Mt $yr^{-1}$ |
| | 11.4 | 36 | 680 Mt $yr^{-1}$ |
| *Delayed Electrification – storage rate* | 8.1 | 505 | 380 Mt $yr^{-1}$ |
| | 8.1 | 367 | 380 Mt $yr^{-1}$ |
| | 8.6 | 50 | 380 Mt $yr^{-1}$ |
| | 8.9 | 36 | 380 Mt $yr^{-1}$ |
| *Net negative – cumulative* | 8.0 | 500 | 4.7 Gt |
| | 8.0 | 360 | 4.7 Gt |
| | 8.4 | 51 | 4.7 Gt |
| | 8.5 | 36 | 4.7 Gt |
| *Net negative – storage rate* | 8.7 | 505 | 465 Mt $yr^{-1}$ |
| | 8.7 | 363 | 465 Mt $yr^{-1}$ |
| | 9.4 | 51 | 465 Mt $yr^{-1}$ |
| | 9.7 | 36 | 465 Mt $yr^{-1}$ |
